# Persistent Organic Pollutants in Serum and Several Different Fat Compartments in Humans

**DOI:** 10.1155/2011/417980

**Published:** 2011-04-10

**Authors:** George W. Yu, John Laseter, Charles Mylander

**Affiliations:** ^1^George Washington University Medical Center, Washington, DC 20037, USA; ^2^Accuchem Laboratories, Richardson, TX 75081, USA; ^3^Department of Statistics, Naval Academy, Annapolis, MD 21401, USA

## Abstract

*Background*. Chemicals that store in lipid-rich compartments have the potential for long-term disruption of metabolic and endocrine processes. Given the evidence that persistent organic pollutants (POPs) also alter systemic metabolic, endocrine, and immune system functions, it follows that elevated chemical concentrations in intra-abdominal fat may alter function, through local chemical signaling, of visceral organs. Despite this potential, there has been little study defining POP concentrations in live human intra-abdominal fat. It is at present uncertain whether POPs distribute equally to all fat compartments, including fat in serum. *Methods*. Seven human subjects scheduled for elective surgery for benign lesions or cancer provided consent for removal of samples of subcutaneous and intra-abdominal fat and/or cancerous tissue. These samples were analyzed for 22 chlorinated pesticides and 10 polychlorinated biphenyl (PCB) congeners by GC/ECD plus GC/MS. *Results*. In only two subjects were the patterns and relative concentrations of PCBs and pesticides about the same in all fat compartments. In the other subjects, there were major differences in levels in subcutaneous as compared to other compartments, but with some higher and some lower. While the pattern of PCBs in the various compartments matched that of the pesticides in some, it was opposite in others. *Interpretation*. These results demonstrate a complicated distribution of PCB congeners and pesticides in various lipid compartments. The difference may reflect various K_ow_s, different rates of metabolism, and/or different lengths of exposure. But the results suggest that contaminant levels in serum or even subcutaneous fat do not necessarily indicate concentrations and patterns in other kinds of adipose tissue.

## 1. Introduction

While adipose tissue has in the past been viewed as a depot involved in passive storage of energy, it is increasingly clear that it is in fact a highly active tissue. It is an endocrine organ, secreting various cytokines and regulating metabolism of other organs. Adipose tissue affects not only body weight, but also insulin sensitivity, atherogenesis, blood pressure, and other physiological functions [1]. The body has several depots of white adipose tissue, of which the major two categories are subcutaneous and visceral, although visceral fat can be divided into subcategories of intraperitoneal, retroperitoneal, and pelvic. Most studies have shown relatively little difference in the fatty acid composition of the various fat stores including fat in serum [2]. However, there is suggestion that this may not be the case in obese individuals [3] and Kishino et al. [4] reported that visceral fat thickness was positively correlated to levels of palmitic and saturate fatty acids in serum, but negatively correlated with levels of linoleic and polyunsaturated fatty acids. There is a growing body of evidence that many of the cytokines and growth factors have depot-specific distribution in human adipose tissue [5–7]. It is known that visceral fat correlates better with the development of insulin insensitivity and the metabolic syndrome than does subcutaneous fat, and this may be related to the demonstrations that there is greater glucose uptake [8] and insulin metabolism [9] in visceral fat.

Persistent organic pollutants (POPs) are highly lipophilic chemicals that bioaccumulate in animal and human fats [1]. POPs includes chemicals such as dioxins/furans, polychlorinated biphenyls (PCBs), chlorinated pesticides, brominated flame retardants, and perfluorinated compounds. Because of chlorine, bromine, or fluoride groups on the hydrocarbon rings or chains, these substances are resistant to degradation both in the environment and in the human body. The half-lives of substances such as DDE, the major metabolite of DDT, and some PCB congeners are of the order of 5–10 years, and because of their presence in almost all animal fat consumed with the diet, the concentrations in the human body tend to increase with age because rates of intake exceed those of metabolism and excretion [10]. While the manufacture and use of most of these chemicals has been prohibited in the US through the Toxic Substances Control Act of 1976 and their manufacture and use on a global scale is outlawed by the Stockholm Convention of the United Nations which entered into force in 2004, these toxic chemicals remain present in the bodies of most humans.

POPs are toxic to human health, being probable human carcinogens, having immunosuppressive activity, causing neurotoxicity, increasing risk of diabetes, cardiovascular disease, and hypertension, and being endocrine disruptive chemicals [11, 12]. Different POPs often have somewhat different health outcomes, and most studies have focused on only single chemicals. However, since essentially all humans are exposed to many different POPs, but perhaps in different relative concentrations, it is important to consider the health effects of chemical mixtures. Multiple chemicals may have additive, less than additive, or synergistic effects [13]. Because POPs are stored in adipose tissue and because the various adipose tissues of the body have somewhat different endocrine functions, it is important to know whether or not POPs distribute equally throughout all adipose tissues and ultimately to determine how levels of these chemicals influence the local adipose tissue. There is some evidence that this may occur as Arsenescu et al. [14] have reported that PCB 77 but not PCB 153 induces adipocyte differentiation and expression and release of proinflammatory cytokines.

This preliminary study of analysis of PCBs and chlorinated pesticides in various adipose tissues obtained from seven human subjects is a first attempt to determine whether the distributions of several PCB congeners and several chlorinated pesticides are the same in different adipose tissues.

## 2. Materials and Methods

Seven human subjects scheduled for elective surgery for either benign lesions or cancer provided informed consent for the surgical team to obtain blood and fat biopsies from several different fat compartments at the time of surgery for analysis of ten PCB congeners and 22 chlorinated pesticides. The study was reviewed and approved by the local Institutional Review Board. Four subjects (3 males and 1 female) presented with renal cell carcinoma, 1 male had a benign renal cyst, 1 male had a benign small intestinal tumor, and 1 male had prostate cancer. All subjects had preoperative CAT scans of the abdomen and pelvis, bone scans, and complete blood metabolic studies. One surgeon obtained the fat samples from all of the subjects during a laparotomy. After blood samples were drawn, the surgeon excised samples of 5 grams or greater from the subcutaneous peripheral fat as well as from one or more intra-abdominal fat stores, including visceral, retroperitoneal, and pelvic fat as well as from the kidney or prostate that was removed at surgery.

**Figure 1:**
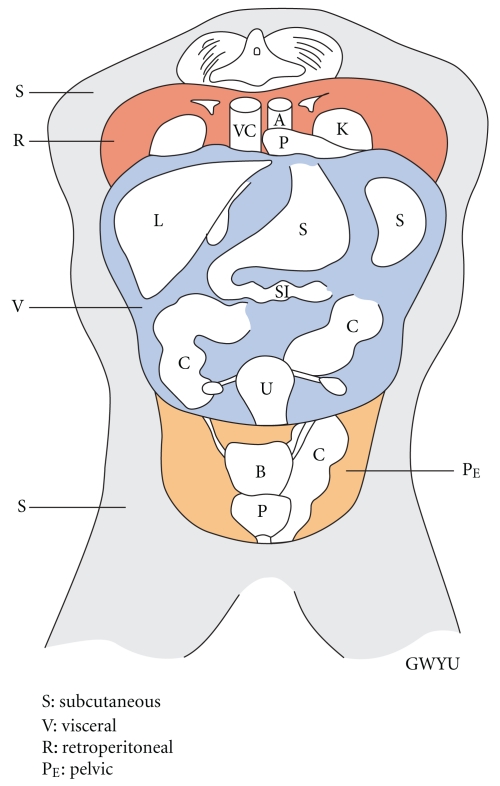
Fat components. Organs are identified as follows: VC: vena cava; A: aorta; P: pancreas; K: kidney; L: liver; S: stomach and spleen; C: colon; U: uterus; B: bladder; P: prostate.

Visceral fat commonly refers to adipose tissue within the peritoneal cavity. In some subjects, visceral fat from three different locations was obtained, the mesenteric fat from around the small and large intestines, omental fat from the folds of the peritoneum that connect the stomach with other abdominal organs, and fat from the Falciform ligament that attaches part of the liver to the diaphragm and abdominal wall. Retroperitoneal fat lies posterior to the peritoneal compartment and is separated from it by the posterior peritoneum. Retroperitoneal fat surrounds the adrenal glands, pancreas, kidneys, and the great vessels as well as large lymphatic channels. The pelvic fat compartment is an inferior extension of the retroperitoneal fat and surrounds the iliac vessels, rectum, bladder, and prostate in males. The pelvic fat samples consisted of fat adjacent to the prostate and rectum. Figure 1 shows the location of the various fat stores as well as the major abdominal organs. 

Serum and fat samples were analyzed by Accu-Chem Laboratories, Richardson, TX. PCBs and chlorinated pesticides were extracted into hexane after the addition of an internal standard (2,2′,3,3′,4,5,5′,6-octachlorobiphenyl). The solvent was then evaporated to dryness and the sample reconstituted and analyzed on a gas chromatograph (Hewlett Packard 5890) equipped with an electron capture detector using a Hewlett Packard 5970 mass spectrometer and a DB5 capillary column. Three sets of standard references were used, one from the national Institute of Standards and Technology and two commercially available reference sets. The level of detection was 0.15 ng/mL and the level of quantitation was 0.3 ng/mL. Twenty three chlorinated pesticides were measured, including hexachlorobenzene (HCB), endrin, *α*, *β*, *δ*, and *γ* hexachlorocyclohexane (HCH), heptachlor, heptachlor epoxide, *α*, and *γ* chlordane, oxychlordane, trans-nonachlor, endosulfan I and II, endosulfan sulfate, aldrin, dieldrin, dichlorodiphenyltrichloroethylene (DDE), dichlordiphenyltrichlorethane (DDT), dichlorodiphenyldichloroethane (DDD), methoxychlor, mirex and endrin aldehyde. Ten PCB congeners were analyzed including PCBs 47 (2,2′,4,4′), 105 (2,3,3′,4,4′), 118 (2,3′,4,4′,5), 137 (2,2′,3,4,4′,5), 138 (2,2′,3,4,4′,5′), 153 (2,2′4,4′,5,5′), 170 (2,2′3,3′4,4′,5), 171 (2,2′3,3′,4,4′,6), 180 (2,2′3,4,4′5.5′), and 183 (2,2′3,4,4′,5′,6). All results are reported as wet weight values, based in part on the basis of the demonstration [15, 16] that lipid adjustment increases the bias in the measurement. The limits of detection were 0.3 ppb in serum, 10 ppb in fat and kidney cancer tissue, and 35.7 ppb in cancerous tissue.

## 3. Results

Table 1 gives the results of pesticide levels in the various samples from the seven subjects, while Table 2 provides similar information for PCB congeners. Of the 23 pesticides monitored, none of the samples showed levels of aldrin, *α* and *γ* chlordane, *δ* and *γ* HCH, endosulfan II, endosulfan sulfate, DDD, DDT, endrin aldehyde, or methoxychlor above the level of quantitation, while for PCB congeners levels of PCBs 47 and 171 were not found in any tissue at concentrations above the levels of quantitation. Therefore, these substances are not included in the tables.

**Figure 2:**
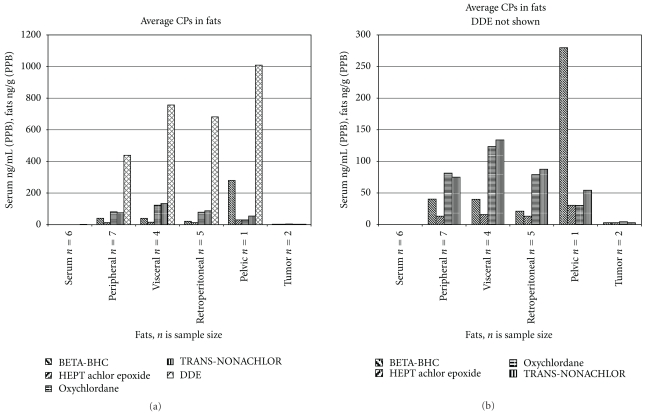
(a) Average levels of elevated selected Chlorinated Pesticides found in serum and fats. (b) Average levels of elevated selected Chlorinated Pesticides other than DDE found in serum and fats. The number of samples taken in the various compartments is the value of *n*.

**Figure 3:**
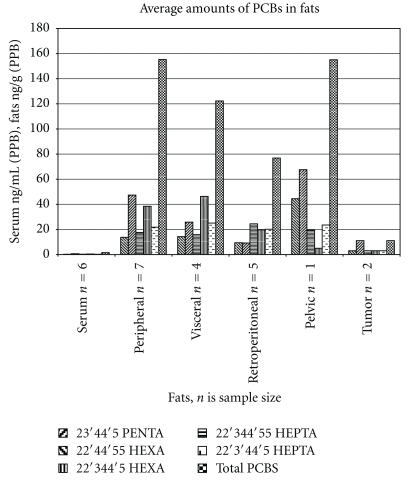
Average levels of PCB and selected components found in serum and fats. The number of samples taken in the various compartments is the value of *n*.

Figure 2(a) shows the average levels of selected pesticides in serum and various adipose tissues.  Figure 2(b) shows the same data without DDE, which is present at much higher concentrations than any other pesticide. This figure clearly shows that there are some differences in the patterns, most striking in the one individual from whom a pelvic sample was obtained. The differences are less clear for those tissues for which more samples were obtained.  Figure 3 shows a similar plot of PCB results.

**Figure 4:**
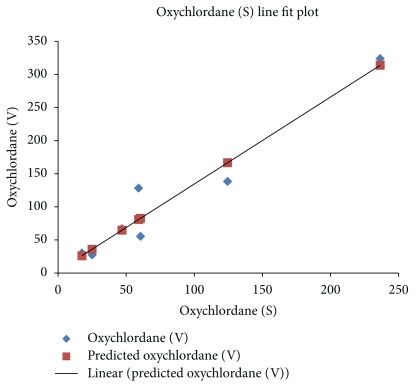
Exploring the relationship between quantities of one toxic chemical found in subcutaneous fat (S) and either visceral fat (V) or pelvic fat. Linear Fit: oxychlordane (V) = 3.0888217 + 1.3144289 oxychordane (S) *R* ^2^ = 0.939, *P* = .0003.

Figure 4 shows a plot of oxychlordane concentration in visceral versus subcutaneous adipose tissues and demonstrates that there is a very close relationship with an *R* ^2^ of 0.939, *P* = .0003.

## 4. Discussion

The results of this investigation show a much more complex picture for the distribution of pesticides and PCBs in various adipose tissue pools than expected. It is usually assumed that in a fasting person (all of these surgical patients were fasting), POPs will be equally distributed in all adipose tissue sites, and consequently one can obtain use serum samples to monitor body burden of lipophilic substances. While serum usually contains somewhere between 1–4% lipid, the assumption is that the POPs concentrations in the serum lipid fraction reflect all adipose tissue. In the past, results were usually reported as lipid adjusted concentrations [17] although reports that lipid-adjustment creates bias [16] have resulted in more reports of wet weight values or using serum lipids as a covariate. Unfortunately, the sensitivity used in the serum analyses in these subjects was not sufficiently sensitive so as to provide much basis for comparison, but there is valuable data from the different adipose tissue sites. 

It is clear that levels of individual POPs in any one individual reflect both the degree and source of exposure, the time since that exposure occurred, genetic differences among individuals in rates of metabolism, body mass index and, if female, number of pregnancies and whether or not the child was breast fed. Most metabolism of POPs is a result of activity of cytochrome P450s, and the ability to metabolize individual POPs varies with structure and genetic susceptibility. For PCBs, for example, congeners with fewer chlorines are in general more easily metabolized than those with more chlorines [18]. Thus, an individual with major exposure in the distant past will show a congener pattern dominated by higher chlorinated congeners. However one would assume that the same pattern would be found in the various fat stores. 

For PCBs, there is reasonably good correlation between serum levels and those in subcutaneous fat for GK, HM, and AS but not MS, GO, and JT. When comparing the different adipose tissues, there is a clear tendency for visceral and retroperitoneal to have a greater percentage of higher chlorinated congeners than subcutaneous fat for GK, GO and JT. However, for ML subcutaneous and visceral patterns were similar but concentrations higher than in retropertoneal fat. MS showed relatively similar patterns, but with concentrations in mesenteric fat considerably greater than in subcutaneous fat, while AS showed higher concentrations in subcutaneous fat. HM, the only subject with pelvic fat, showed relatively similar concentrations for PCBs 118, 138, and 170, but high levels of 153 in pelvic but not subcutaneous and of 180 in subcutaneous but not pelvic fat. 

The patterns among the pesticides also showed surprising variability. DDE was present in the highest concentration in most samples, but was below the detection limit in subcutaneous fat in GK and JT in spite of being present at high concentrations in serum and other adipose tissues. In all other subjects, DDE concentrations were similar in the different adipose tissues. Dieldrin, heptachlor epoxide, oxychlordane, and transnonachlor were present in most adipose samples and had relatively similar distributions in all fat stores. *β*HCH was present in comparable amounts in most samples that contained measureable levels, and *α*HCH was found in only a few samples. Interesting *γ*HCH (lindane) was not detected in any sample. Mirex was found in serum at a relatively high concentration in GK, but was not present in any adipose tissue, and similar situation was found with heptachlor in ML. These results suggest a very recent exposure to these two POPs that has not yet equilibrated with adipose tissue. 

When comparing general levels of PCBs to those of the pesticides, distributions were similar for ML, HM, and GO. However for AS and JT, PCBs were at higher concentrations in subcutaneous fat, but pesticides were at higher concentration in visceral and retroperitoneal fat. GK showed few pesticides in subcutaneous fat relative to other stores. For MS levels of PCBs were greater in subcutaneous than visceral fat, but levels of pesticides were approximately equal in the two stores.

Only one cancerous tissue (kidney) gave detectible levels of POPs, and it contained PCB 153 and oxychlorodane at relatively low concentrations, but no other POPs. These two POPs were present in the highest concentration in subcutaneous fat. Since the lipid content of a tumor would be expected to be significantly less than adipose tissue, the relative wet weight results are not surprising.

It is unlikely that the variable results seen in levels of both PCB and pesticides are due to chance or to faulty chemical analysis. There is always some variability in analysis of POPs, but usually this is not more than 20%. The differences seen are too large to be due to experiment variability. 

There are a variety of possible explanations for these surprising results. There are reports that there are large differences in the average fatty acid composition between individual subjects, as well as some differences in composition of different adipose tissue sites within subjects [19]. If this is the case, it is possible that the lipid composition of the various adipose tissue stores is reflected in different solubility of individual POPs in different depots even in the same individual. There is not a lot of information on lipid composition. Even subcutaneous fat taken from different parts of the body has been found to show between 2–10% variation [2, 20]. There is some information suggesting that subcutaneous and visceral fats differ more, with saturated fats higher and monounsaturated fat lower in visceral than subcutaneous fat [3]. 

Since P450 enzymes are found in almost all cells, not just liver, it is conceivable that there is different metabolism in different fat depots. Certainly there is evidence that different depots express different endocrine factors. However, unless the POPs were bound in some fashion, it seems unlikely that even if there were differences in local metabolism that there would not be a relatively rapid redistribution. 

Botella et al. [21] reported on organochlorines pesticides in serum and adipose tissue of Spanish women. They reported six POPs where serum levels correlated relatively well with those in adipose tissues, but no clear relationship with the other nine POPs. The mean adipose/serum ratios varied from 2.85 for endosulfan-ether to a high of 150.68 for DDE and 159.47 for endosulfan II. They questioned the common assumption that serum concentrations are indicative of those in adipose tissues. Our results are consistent with their observations and indicate that the same surprising relationship may also occur with different PCB congeners.

## 5. Limitations

There are major limitations to this study because of small sample size, lack of measurement of the lipid content of tissues, and the relatively low sensitivity of the analytical methods. However, the difficulty in obtaining samples and the lack of any comparable study elsewhere in light of the overall importance of the question of how different POPs distribute in different adipose tissue depots is justification for publishing these results. Replication is certainly needed to confirm the overall conclusion that POPs do not always distribute equally among various lipid compartment in the human body.

## 6. Conclusions

Contrary to expectations that patterns of various pesticides and PCB congeners were not as similar as expected when comparing levels in serum, subcutaneous, visceral, retroperitoneal, and pelvic fat. These results indicate that care should be taken when assuming that serum levels of POPs reflect patterns found in lipid stores throughout the body.

## Figures and Tables

**Table 1:** Pesticides (ppb wet weight) in various adipose tissues and serum. (— indicates values below the limit of detection).

Pesticides (ppb wet weight)												
GK	DDE	Dieldrin	Endosulfan 1	Endrin	HCB	Heptochlor	Heptochlor epoxide	*α*HCH	*β*HCH	Mirex	Oxychlordane	Trans-nonachlor
Serum	1.3	—	—	—	—	—	—	—	—	1.6	—	—
Subcutaneous	—	—	—	—	31.8	—	—	—	—	—	46.9	—
Visceral												
Meseuteric	351.7	15.4	—	—	—	—	11.2	19.3	34.5	—	63.3	54.4
Omental	277.6	23	—	—	—	—	—	—	—	—	54.4	46.4
Falciform ligament	436.5	15.8	94.9	—	—	—	11.5	—	17	—	83.3	70.3
Retroperitoneal	427.6	25.4	—	—	—	—	12.3	—	20.7	—	87.3	71.9

ML	DDE	Dieldrin	Endosulfan	Endrin	HCB	Heptochlor	Heptochlor epoxide	*α*HCH	*β*HCH	Mirex	Oxychlordane	Trans-nonachlor
Serum	0.4	—	—	—	—	0.3	—	—	—	—		—
Subcutaneous	276.2	Trace	—	—	Trace	—	13.8	—	Trace	—	25	17.3
Visceral	222.1	Trace	Endosulfan 1	—	Trace	—	10.9	—	Trace	—	27.4	15.9
Retroperitoneal	117.8	—	—	—	Trace	—	Trace	Trace	Trace	—	10.3	—
Gerota's	174.3	—	—	—	Trace	—	—	—	Trace	—	10.9	—

HM	DDE	Dieldrin	Endosulfan 1	Endrin	HCB	Heptochlor	Heptochlor epoxide	*α*HCH	*β*HCH	Mirex	Oxychlordane	Trans-nonachlor
Serum	1.5	—	—	—	—	—	—	—	—	—	—	—
Subcutanrous	647.5	23.1	—	—	51.2	—	11.4	13.8	115.2	—	17.5	30.2
Pelvic	1009.1	—	—	—	141	—	30.6	11	279.8	—	30.2	54.4

MS	DDE	Dieldrin	Endosulfan 1	Endrin	HCB	Heptochlor	Heptochlor epoxide	*α*HCH	*β*HCH	Mirex	Oxychlordane	Trans-nonachlor
Serum	0.76	—	—	—	—	—	—	—	—	—	—	—
Subcutaneous	579.2	16	—	—	—	—	23.9	—	15.5	—	60.5	144.7
Visceral-(Mesenteric)	466.6	48.2	20.3	—	—	—	23.2	—	—	—	55.4	149.4

AS	DDE	Dieldrin	Endosulfan 1	Endrin	HCB	Heptochlor	Heptochlor epoxide	*α*HCH	*β*HCH	Mirex	Oxychlordane	Trans-nonachlor
Serum	3.1	—	—	—	0.3	—	—	—	Trace	—	0.3	0.4
Subcutaneousl	743.7	—	—	—	—	—	—	—	104.4	—	236.4	194
Visceral	960.6	16.6	—	—	—	—	20.8	—	140.9	—	323.9	258.6

GO	DDE	Dieldrin	Endosulfan 1	Endrin	HCB	Heptochlor	Heptochlor epoxide	*α*HCH	*β*HCH	Mirex	Oxychlordane	Trans-nonachlor
Serum	0.3	—	—	—	—	—	—	—	—	—	—	—
Subcutaneous	819.3	—	—	—	—	—	14.2	—	34.3	—	124.4	130.8
Visceral	698.1	13.5	—	—	—	—	15.4	—	36.3	—	138.4	139.4
Retroperitoneal (Perirenal)	700.4	—	—	—	—	—	12	—	31.5	—	104.3	112.4

JT	DDE	Dieldrin	Endosulfan 1	Endrin	HCB	Heptochlor	Heptochlor epoxide	*α*HCH	*β*HCH	Mirex	Oxychlordane	Trans-nonachlor
Serum	6.3	—	—	—	—	—	—	—	—	—	0.3	—
Subcutaneous	—	—	—	—	—	—	12.1	—	—	—	59.1	—
Visceral												
Mesenteric	2147.71	34	—	—	—	—	34.7	—	48.3	—	189.9	246.2
Omental	1170.3	14.9	198.2	—	—	—	12.5	—	19.8	—	66.6	120.7
Retroperitoneal (Gerota's)	1426.4	21.7	162.5	—	—	—	21.9	—	25.4	—	114.3	162.6
Tumor (kidney)	—	—	—	—	—	—	—	—	—	—	12.2	—

**Table 2:** PCBs in various fat compartments (ppb wet weight) (— indicates values below the limit of detection).

	105	118	137	138	153	170	180	183	Total
GK									
Serum	—	—	—	—	0.6	0.4	0.6	—	1.6
Subcutaneous	—	—	—	21.5	39.1	18.8	32.1	—	111.5
Visceral									
Mesenteric	—	—	—	—	—	24.5	35.3	—	59.8
Omental	—	—	—	—	—	21.5	33.5	—	55
Falciform ligament	—	—	—	—	—	17.8	26.8	—	44.6
Retroperitoneal	—	—	—	—	—	22	42.9	—	64.9

ML									
Serum					tr				
Subcutaneous	—	11.7	—	19.1	43.2	11.7	23.0	—	108.7
Visceral	—	Trace	—	19.7	50.6	Trace	—	—	94.4
Retroperitoneal	—	Trace	—	Trace	21.4	—	—	—	32.6
Gerota's	—	Trace	—	—	34.1	Trace	—	—	34.1

HM									
Serum	—	—	—	—	—	—	0.4	—	0.4
Subcutaneous	—	20.9	—	18.5	—	28	56.7	—	124.1
Peloic	—	44.4	—	19.6	67.6	23.5	—	—	155.1

MS									
Serum	—	—	—	—	0.37	—	0.33	—	0.7
Subcutaneous	—	30.9	—	16.4	32.7	12.6	22.3	—	114.9
Visceral-mesenteric	—	56.4	—	42.3	92.1	39.2	60.1	17.4	317.5

AS									
Serum	—	0.3	—	0.4	0.7	—	0.4	—	1.8
Subcutaneous	—	21.7	—	31.7	50.2	18	35.2	—	156.8
Visceral	—	—	—	24.2	—	13.7	27.8	—	65.7

GO									
Serum	—	—	—	—	0.4	—	0.3	—	0.7
Subcutaneous	—	—	—	—	—	25	43.7	—	68.7
Visceral	—	—	—	—	—	28.7	45.3	—	74
Retroperitoneal (Perirenal)	—	—	—	—	—	27.9	42.7	—	70.6

JT									
Serum	—	0.6	—	1	1.4	0.4	0.8	—	4.2
Subcutaneous	13.9	—	12.3	84.8	158	38.3	75.9	18.8	402.3
Visceral									
Mesenteric	—	—	—	—	—	35.1	80.7	—	115.8
Omental	—	—	—	—	—	39.8	89.7	—	129.5
Retroperitoneal (Gerota's)	—	—	—	—	—	30.9	68.7	—	99.6
Tumor (kidney)	—	—	—	—	19.2	—	—	—	19.2
